# The Paleolithic Diet

**DOI:** 10.7759/cureus.34214

**Published:** 2023-01-25

**Authors:** Annapoorna Singh, Daulath Singh

**Affiliations:** 1 Internal Medicine, St. Francis Hospital, University of Kansas Health System, Lawrence, USA; 2 Internal Medicine/Hematology-Oncology, Stormont Vail Health, Topeka, USA

**Keywords:** hunter-gatherer, ancestor diet, anciet diet, paleo, paleolithic diet

## Abstract

The promotion of healthy diets is likely one of the most cost-effective strategies for preventing a wide range of disorders, including cardiovascular disease, obesity, hypertension, and type 2 diabetes. The majority of present non-communicable chronic diseases are attributed to civilization, an abundance of food, and a lack of physical activity. According to the purported lifestyles of early humans, the paleolithic diet (PD) encourages the intake of wild animal and plant foods. In recent decades, the paleolithic diet has grown in popularity, particularly among younger populations and those with cardiometabolic syndrome and other chronic diseases. Due to the nutrition profile of the paleolithic diet, historical studies have postulated that hunter-gatherers (HGs) have slender physiques and were physically fit and devoid of chronic ailments such as cardiovascular diseases (CVDs). This review highlights the composition/constituent of the paleolithic diet along with an emphasis on current studies and evidence on the effectiveness of the paleolithic diet in mitigating chronic diseases.

## Introduction and background

Nutrition plays an integral part in disease prevention and management, although often overlooked. There have been many food trends over the last several decades ranging from fewer carbohydrates to ketogenic diet, intermittent diet, vegan diet, and paleo diet, among others. There have been conflicting opinions about each diet, causing confusion among general people regarding the right diet. The right diet should include a good proportion of fresh leafy vegetables, fruits, nuts containing healthy fats, and seafood, and consumption of lean meat and red meat in moderation with limitation and exclusion of highly processed food, refined sugars, and calorie-dense food. The right diet should not be restrictive of any particular food items, but rather, emphasis should be placed on moderation in quantity. This review highlights the origination of the paleolithic diet (PD), its composition, and its health consequences.

## Review

The Miocene to the early Pleistocene era, Paleolithic era, Neolithic era, and Industrial Revolution are the four phases of the evolution of the human diet [1]. According to some writers [2], primitive *Homo sapiens* were omnivores who ate much more vegetables than meat. Others [3] claim that animals were consumed more significantly than vegetables, especially lean meat and fish. Tribes that live today as hunter-gatherers (HGs) and in conditions similar to the Paleolithic period include 30% animal-origin products and 70% vegetable-origin products, and they feed almost entirely on animal items if they live in extremely cold areas [4,5], implying that animal-origin food was a part of the diet of primitive *Homo sapiens*. According to a dental topography study, successive *Homo species* emphasized tougher and more elastic diets, maybe including meat. The agricultural era developed about 10,000 years ago, ending the Paleolithic era. Grains and legumes were essential meals, dairy products and vegetable oils such as olive oil became accessible, and the diet was high in fiber, protein, and plant sterols. However, the Industrial Revolution revolutionized human eating; it brought convenient meals such as canned meats, condensed soups, hydrogenated oils, and refined cereals such as white bread [1].

Comparing HGs and agricultural worker populations simultaneously revealed that the former had a greater lifespan and fewer degenerative and other illnesses such as anemia and osteoporosis. These illnesses presumably arose due to the agricultural revolution diet containing fewer food items, primarily grains, and cereals with less absorbable nutrients, resulting in potential nutritional inadequacies [3,6]. According to critics of the paleolithic diet, hunter-gatherers had a low risk of developing degenerative, age-related illnesses since they perished early [7]. However, studies of young people from industrialized societies and modern hunter-gatherer societies revealed that the former had significantly higher rates of obesity, hypertension, insulin resistance, and atherosclerosis than the latter, who also possessed greater muscle strength and aerobic capacity [3]. Presently, vegetables are grown using chemical pesticides, and meat contains a significant proportion of saturated fat.

Furthermore, a substantial proportion of the population does not engage in physical activity, and the population has expanded exponentially. Therefore, the lifestyle that most of the human population lives in today does not correspond to the diet and metabolism for which it was designed [7]. In addition, there are variances regarding the content of meat. Today’s various forms of meat are generated from domesticated, restricted animals fed concentrated diets. These settings bestow a significantly higher concentration of lipids in the meat compared to the core of wild animals, which Paleolithic humans consumed. Recent research demonstrates that wild animal meat contains less than 4% lipids, whereas domesticated meat contains between 25% and 30% lipids. In their lean tissues (muscles), wild animals have large amounts of unsaturated fatty acids (FAs), which are suitable for health. In contrast, confined animals have high concentrations of saturated fatty acids, which are detrimental [7].

Humans have bigger brains than other primates [8,9]. Understanding the human brain’s composition and biochemistry is thus essential. Brain long-chain polyunsaturated fatty acid (PUFA) (LCP) profiles are unique, with about equal levels of arachidonic acid (AA) and docosahexaenoic acid (DHA) [10]. Thus, the structural need for AA and DHA seems to be constant and is required for brain growth and encephalization [11]. AA can be found in plant foods; however, DHA is mainly obtained from seafood, mackerel, salmon, and trout, being good sources [1]. Gut morphology studies [12,13] support the introduction of animal foods, at the expense of vegetable foods, in the diet of early *Homo*. An ape’s colon (45%) dominance indicates adaptation to a diet high in bulky plant material such as fiber and woody seeds.

In contrast, the small intestine’s dominance of the human gut (56%) indicates adaptation to a highly digestible food, implying a structural similarity to carnivores rather than folivorous or frugivorous animals. From *Ardipithecus* and *Australopithecus* to early *Homo*, improved nutrition correlated with increased height, brain size, and metabolic activity [11]. Marine, lacustrine, and riverine animals supplied meat to modern *Homo sapiens* and may have affected brain ontogeny [14].

**Figure 1:**
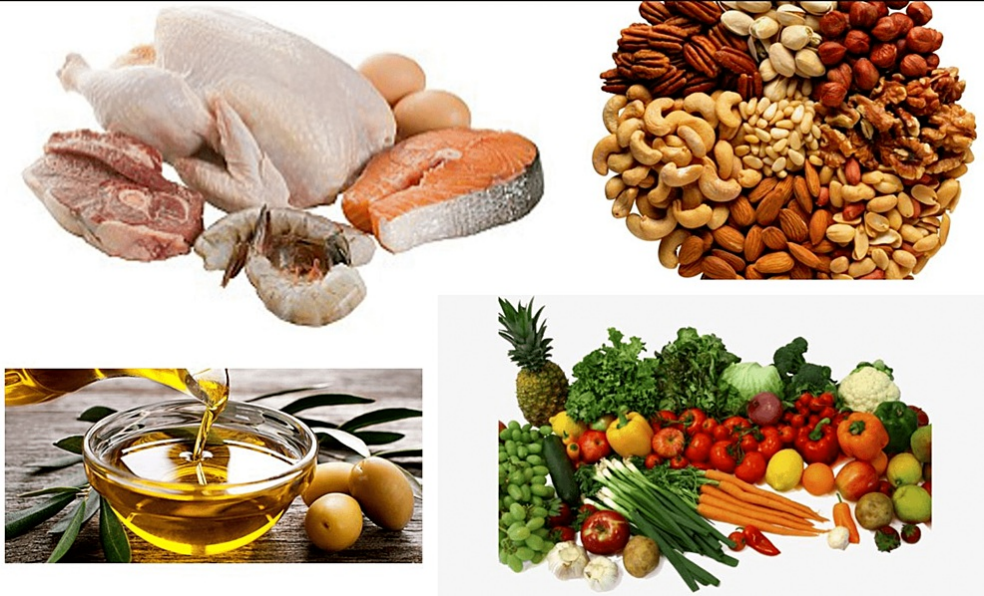
The paleolithic diet The paleolithic diet contains lean meat, nuts, olive oil, fresh vegetables, and fruits.

Therefore, the present epidemic of civilizational illnesses is attributed to the mismatch between our contemporary food and our paleolithic DNA [11]. In other words, “we are what we eat” [15,16]. Unfortunately, today’s western diets are heavily processed, with dairy products, cereals, refined sugars, refined vegetable oils, and alcohol particularly prevalent [17]. Therefore, the paleolithic diet (PD) was calculated to include 37% protein, 41% carbohydrate, and 22% fat (Figure 1).

In comparison, the average American diet contains about half its daily calories as carbohydrates, one-third as fat, and 15% as protein [1]. The paleolithic diet was generally richer in vitamins and minerals than the standard American diet. Processed foods account for approximately 77% of dietary salt in the American diet [1]. The ratio of n-6 fatty acids (n-6 FAs) to n-3 fatty acids (n-3 FA) has also shifted; today’s diet has 10 times more n-6 FAs than n-3 FAs, while HGs ate a 1:1 ratio of n-6 to n-3 FAs [18]. Eicosanoids from n-6 FAs are pro-inflammatory and promote platelet aggregation, while those from n-3 FAs have oppositive effects [19].

Given the supposed advantages of the PD, it is not unexpected that researchers have undertaken experiments, including the implementation of PD, to evaluate its impact on health outcomes. Osterdahl et al. [20] explored the effects of the PD over a three-week period on CVD risk factors in healthy human volunteers. The results showed decreases in mean weight, body mass index, waist circumference, and systolic blood pressure. Similarly, Lindeberg et al. [21] also examined the impact of a PD in comparison to the Mediterranean diet over a 12-week period in those with ischemic heart disease and type 2 diabetes. The results revealed significant improvements in glucose tolerance as well as weight and waist circumference reduction. Thus, PD is shown to be better than the current proven healthiest diet for humans, the Mediterranean diet. Nevertheless, there are other arguments opposing Paleolithic diets. The paleolithic diet is rich in animal protein from sources such as wild animals and fish. However, wild animal meat is no longer widely available. Moreover, although hunter-gatherers ate a lot of animal protein, they also ate more monounsaturated and polyunsaturated fatty acids, n-3 fatty acids, and fruits and vegetables. All of these elements are believed to mitigate the negative consequences of a high-protein diet [22]. Another potential risk with consuming a Paleolithic diet in our current world is the possibility of higher environmental toxin exposure when increasing fish consumption to paleolithic diet levels. Methylmercury is a huge concern when consuming large amounts of fish. The Environmental Protection Agency’s mercury reference dose is 0.1 mg per kilogram of body weight each day [23].

Deviation from our ancestor’s diet

According to the preceding reasoning, our ancestors’ diet included meat and seafood, representing 30%-35 % of the diet. Whether omnivores or carnivores, the diet was not entirely devoid of meat/seafood compared to our modern vegan or vegetarian diet. Today’s civilized diseases are the result of the consumption of processed, highly refined foods, sweets, and fats and increased consumption of red meat; thus, one can argue that the solution should be to eliminate the consumption of processed/refined foods and decrease meat consumption, along with nuts, vegetables, and fruits, to have a more balanced diet, rather than to eliminate animal sources as is espoused. The optimum nutritional composition for healthy health should be balanced. The human genome, which evolved over millions of years, has developed on this equilibrium, which would be present in foods eaten by Paleolithic and perhaps pre-Paleolithic predecessors. Increased productivity per unit of the land area resulted in a net increase in population. Life expectancy in “civilized” societies rarely exceeded 25 years from the late Neolithic to the late 18th century. Increases in life expectancy can be traced back to the onset of the Agricultural Revolution, during which numerous areas, including sanitation, food production and processing, energy generation, housing, and transportation, experienced substantial advancements.

Certain nutrients, such as DHA, vitamin B12, and heme iron, are exclusively found in animal-based products. Collagen is similarly synthesized spontaneously in the body by combining amino acids (proline from eggs and glycine from pork and chicken), mainly derived from animal sources; it is essential for joint health and skin suppleness and cannot be obtained from plant foods. It also depletes with time. In addition, it aids in osteoarthritis treatment and regulates blood pressure and blood sugar levels. Likewise, chondroitin (found in animals’ connective tissues) and glucosamine (shellfish) are also necessary for bone and joint health. These nutrients should ideally be obtained through diet alone, and current guidelines do not recommend routine supplementation. However, because vegans eat no animal products and are typically younger, supplementing these nutrients should be emphasized and encouraged to maintain bone health in the long run.

## Conclusions

The increasing prevalence of chronic diseases such as cardiovascular disease, obesity, and type 2 diabetes necessitates the implementation of appropriate nutrition choices and guidelines. Because our predecessors were essentially free of these civilized diseases, it is not a stretch to conclude that we should focus on adopting a diet similar to theirs, and in fact, there has been a rise in interest in the advantages of ancestral diets. In spite of the difficulty of adopting a paleolithic diet identical to that of our ancestors in the modern day, it may be possible to adopt variants that are comparable. Emphasis should be placed on the inclusion of lean meat, nuts, extra virgin olive oil, and fresh fruits and vegetables, whereas ultra-processed foods abundant in unsaturated fats, sodium, and refined sugar should be eliminated. Physical activity is of the utmost importance to prevent the storage of extra calories as fat, which is a risk factor for several chronic diseases. Currently, we are moving in the exact opposite direction as our forefathers, consuming an excessive amount of highly processed foods that are nearly devoid of key nutrients and vitamins and engaging in almost little to no physical activity.
